# Pediatric Autoimmune Neuropsychiatric Disorders Associated With Group A Streptococci: Etiopathology and Diagnostic Challenges

**DOI:** 10.7759/cureus.27729

**Published:** 2022-08-06

**Authors:** Andrada Hutanu, Lalitha N Reddy, Janice Mathew, Chaithanya Avanthika, Sharan Jhaveri, Nayanika Tummala

**Affiliations:** 1 Trauma and Orthopaedics, King's College Hospital NHS Foundation Trust, London, GBR; 2 Pediatrics, ACS Medical College, Chennai, IND; 3 Pediatrics, Jubilee Mission Medical College and Research Institute, Thrissur, IND; 4 Medicine and Surgery, Karnataka Institute of Medical Sciences, Hubli, IND; 5 Medicine, Smt. NHL Municipal Medical College, Ahmedabad, IND; 6 Internal Medicine, Gitam Institute of Medical Sciences and Research, Vishakapatnam, IND

**Keywords:** neuropsychiatry symptoms, pandas and ocd, pediatric autoimmune neuropsychiatric disorders associated with streptococcal infections, group a β hemolytic streptococci, children disease

## Abstract

Pediatric autoimmune neuropsychiatric disorders associated with streptococcal infections (PANDAS) have attracted a lot of interest and discussion since it was originally characterized in 1998. The role of streptococcal infection in children with abrupt-onset obsessive-compulsive disorder (OCD) and new-onset tics, the natural history of this entity, and the role of symptomatic and disease-modifying therapies, such as antibiotics, immunotherapy, and psychoactive drugs, are still unresolved issues. Alternative therapies for acute-onset OCD have been developed based on this postulated pathophysiology, including antibiotics and immunomodulatory therapy. The literature on PANDAS therapy is varied but there is no clinical consensus on the treatment of choice.

While there is no convincing evidence for the autoimmune rationale for PANDAS, given the increased attention to this entity and the apparent growth in usage of this diagnostic category, it is critical to address concerns about the condition's diagnosis, treatment, and pathogenesis.

We conducted a multi-language literature search on Medline, Cochrane, Embase, and Google Scholar for a period spanning until October 2021. The following search strings and Medical Subject Heading (MeSH) terms were used: “PANDAS,” “Group A Streptococcus,” “OCD,” and “tics.” We explored the literature on PANDAS in terms of its epidemiology, pathophysiology, the role of group A streptococcal infection, associated complications, and prophylactic and treatment modalities.

We examined current working definitions of PANDAS, analyzed differential diagnoses, and published pieces of evidence for therapies associated with this entity, with a view to proposing a therapeutic strategy for children with acute symptoms that meet PANDAS criteria, in this review article.

## Introduction and background

PANDAS, which stands for pediatric autoimmune neuropsychiatric disorders associated with streptococcal infections, has sparked a lot of interest and debate over the last century [1]. In theory, the connection between group A streptococcus and the impact on the brain is believed to be due to an abnormal response mediated by antibodies. This results in brutal neuropsychiatric manifestations in children with no history of such comorbidities [2]. More specifically, the changes can have obsessive-compulsive symptoms, or occur in the form of uncontrollable movements or speech, such as tics. Apart from the aforementioned manifestations, the symptoms may impact the usual behavioral attitudes of children as well [3].

A few studies have shown PANDAS to be related to an autoimmune disturbance of the mother of the child, which is triggered by a recent streptococcal infection [4]. In terms of the microbial component preceding the brain changes, the site of infection can vary, and consequently the pathologies: from pharyngitis or sinusitis to scarlet fever [2]. On the other hand, some researchers have suggested that PANDAS patients are more prone to streptococcal infections, while simultaneously belonging to the psychiatric patient spectrum. In other words, group A beta-hemolytic streptococcal (GABHS) infections are the only precipitating factors for neurological manifestations, including Tourette's syndrome [5,6].

All things considered, the controversy over PANDAS has equally affected doctors, children, and their families significantly [7]. Concerns and intense emotions of the family members whose children are diagnosed with this phenotype have received significant attention on social media and are well-documented in the news [8]. The reasons behind PANDAS having a great impact on so many levels and populations include a wide range of clinical diagnostic challenges, treatment options that are under discussion, and long-term complications, on top of the fact that none of them has benefited from a clear and standard conclusion [1]. In addition, the autoimmune disease has gained even more attention at a time when researchers have failed to indicate a precise rate of prevalence among children already suffering from obsessive-compulsive symptoms or tics for a long time. Not only have the patients expressed confusion regarding their condition, but the science has also been losing credibility due to its unsuccessful attempts at finding any particular biomarkers for the disorder [9].

Despite the fact that the first cases were registered more than 50 years ago, healthcare professionals appraise PANDAS as a new condition because of the many unknown facts associated with it [3]. Starting with the risk factors, it is scarcely hypothesized that either recurrent GABHS, a history of rheumatic fever, or any other autoimmune disorder in the family plays an important role in determining the rapid onset of neuropsychiatric disturbances [2]. The underlying pathophysiology is not fully understood; nevertheless, scientific professionals blame the cross-reaction of antibodies against group A streptococcus to be responsible for interacting with antigens located in the basal ganglia [10].

Furthermore, even though the diagnosis appears streamlined because of the sudden changes, doctors tend to overlook PANDAS due to differentials overlapping with the disorder [3]. Clinicians have recently pointed out incomplete delineations of the diagnostic criteria from the previous decade [9]. In contrast to the noticeable shift in personality or involuntary motions, it takes a detailed physical examination and a comprehensive history for the doctor to accurately diagnose and differentiate the disease from other very common conditions [3]. However, owing to the nature of the signs, they have classified the manifestations as “major” disturbances [2].

Concerning treatment, the medical community is still conducting trials, ranging from antibiotic therapy to embracing a more immunological approach or delivering psychoactive medications, yet each of them is posing significant questions [1]. Delayed or no treatment, perhaps due to diagnostic obstacles, can lead to damages later in adult life, including a higher rate of occurrence of tics and obsessive-compulsive syndromes. In light of this, choosing the optimal therapy is critical and a matter of urgency not only for doctors but for researchers too [3]. So far, PANDAS has been considered an individual entity among autoimmune disorders, mainly due to the ambiguous relation between GABHS and neuropsychiatric symptomatology, as a result of insufficient explanations or scientifically unproven pieces of evidence to date [10,11].

## Review

Pathophysiology of PANDAS and the role of group A streptococci

The most popular rationale concerning the pathophysiology of PANDAS is the autoimmune concept by the mechanism of “molecular mimicry”, wherein antibodies targeted against streptococcal proteins act against those found in the basal ganglia of the central nervous system (CNS) due to a structural similarity [12].

Studies supporting antineuronal antibodies have been documented. The presence of anti-basal ganglia antibodies in a cohort with Sydenham's chorea (SC) and subsequently in another group of children with neuropsychiatric/movement disorders has been documented [12]. Other studies have additionally described the presence of other antibodies targeted against dopamine (D1/D2) receptors, beta-tubulin receptors, and Lysoganglioside (lyso-GM1) receptors along with increased calcium/calmodulin-dependent protein kinase II (CaMKII) activity [13,14]. While some studies are in favor of this hypothesis, others have not shown any endorsement [15-17]. These antibodies are collectively called the “Cunningham Panel” and have been tested individually. They were proven to be indecisive, as no significant difference was observed between children with and without pediatric acute neuropsychiatric syndrome [18].

When MRI was done on the group in the study by Swedo et al. [19], in the initial course of the disease, it showed enlargement of caudate, putamen, and globus pallidus, which was attributed to local inflammation particularly affecting these regions in the CNS [20].

Animal models have also been used to strengthen the diagnostic criteria of PANDAS. Studies have shown animals to exhibit similar behavioral patterns as seen in PANDAS after being injected with GABHS [21]. Subsequent transfer of this sera to naive rodents/mice has also shown a similar change in behavioral/motor patterns, further supporting these findings [22,23]. However, another study demonstrated that human sera (from PANDAS patients) injected into rodents did not exhibit the same pattern [24].

Further cases of animal studies have shown sera from well-established PANDAS patients binding to cholinergic interneurons in the striatum of mice, and thereby being incapable of doing the same following treatment with intravenous immunoglobulin (IVIG) [25]. Moreover, the treatment response of PANDAS cases to antibiotics and immunomodulatory therapy further supports the autoimmune rationale [26].

A different rationale has been proposed in the study by Quagliariello et al., wherein it was found that streptococcal infection altered gut microbial communities by selecting strains that are normally associated with gut inflammation and activation of the immune response. This was found to persist even after the resolution of infection. They were indirectly found to decrease the synthesis of metabolites in the brain such as tyrosine and dopamine. This altered microbiome may thus have an impact on behavioral changes seen in PANDAS [27].

Another component that may be recognized with regard to the pathophysiology of PANDAS is genetic susceptibility. Studies using interviews to ascertain the risk of autoimmune disorders have found a greater prevalence among mothers with children who have been diagnosed with obsessive-compulsive disorder (OCD)/tics as compared to other women; however, there were limitations due to recall bias and having to rely on oral reports without medical evidence for back-up [28].

There have also been reports suggesting that PANDAS is the effect of a miscellaneous immune dysregulation of regulatory T cells, cytokines, immune-associated genes, cerebrospinal fluid (CSF) oligoclonal bands, and immunoglobulins [29]. Though these theories have been put forward, multiple studies have disproven the same and further studies will be required to ascertain the exact mechanism and immune biomarkers that can be attributed to this relatively new clinical entity of PANDAS.

Clinical features

PANDAS patients experience a rapid onset of symptoms or worsening of OCD symptoms or tics, which may develop along with or shortly after a group A streptococcal infection (which includes pharyngitis, impetigo, etc.), followed by a relapsing-remitting episodic course.

Neuropsychiatric Features

These include a variety of additional symptoms and comorbidities such as severe separation anxiety and other anxiety disorders, motor hyperactivity, abnormal movements, restlessness, sleep disturbances, hallucinations, urinary frequency and urgency, difficulty concentrating, mood swings, major depression, and loss of academic abilities [30]. These may be similar to the presentation of other conditions such as OCD and Tourette's syndrome.

Cardiac Involvement

In a study by Murciano et al., similar autoimmune pathophysiology between PANDAS and rheumatic carditis prompted them to look further into the possible cardiac involvement in children with PANDAS. It was found that a proportion of children with PANDAS had been auscultated with a cardiac systolic murmur and another segment of children had echocardiography abnormalities like a patent foramen ovale (PFO) and ECG abnormalities involving sharp ‘T’ waves and ventricular pre-excitation in a child with a known case of Wolff-Parkinson-White (WPW) syndrome. High ASO titers were not found to be associated with cardiac abnormalities. Based on this, a cardiology screening at the onset and a cardiology follow-up at least once a year after puberty have been recommended [31].

Otorhinolaryngology Symptoms

In the study by Cocuzza et al., a majority of children diagnosed with PANDAS were affected with effusive otitis media closely followed by respiratory sleep disorder. Other pathologies may have included rhinosinusal pathology, sensorineural hearing loss, and temporomandibular joint disorders [32].

Diagnostic protocols and evaluation

PANDAS is diagnosed clinically based on presenting symptoms meeting certain diagnostic criteria (Table 1).

**Table 1:** Diagnostic criteria of PANDAS* *[2] PANDAS: pediatric autoimmune neuropsychiatric disorders associated with streptococcal infections

	Diagnostic criteria
1	Presence of obsessive-compulsive disorder, tic disorder, or both
2	Pediatric-onset between 3 years and onset of puberty
3	Acute, severe onset with relapsing and remitting episodes
4	Temporal relationship between symptom onset and exacerbation and group A beta-hemolytic streptococcal infections
5	Association with neurological abnormalities such as hyperactivity, and unusual jerky movements that are not in the child’s control

Currently, there are no definitive diagnostic lab tests for PANDAS. But all patients who have the above symptoms should undergo initial tests: complete blood count, liver function test, renal function test, CRP, ESR, urine routine, and metabolic panel, and for streptococcal infection, throat swab, or anti-streptolysin-O levels or anti-DNase B level [3,33]. Throat culture positivity remains even after one year of infection and streptococcal antibodies remain elevated for more than a year in over 50% of children indicating either carrier state or active infection, and it does not provide any conclusive evidence for recent streptococcal infection [34]. Other lab indicators include low vitamin D [35], low serum ferritin, and low iron levels detected, raised antithyroid antibodies, and elevated anti-A carbohydrate (anti-ACHO) antibodies [36].

Since the pathophysiology involves autoimmune mechanisms in PANDAS, a commercially available test called Cunningham Panel measuring autoantibodies to dopamine receptors D1 and D2, tubulin, lysoganglioside-GM1, and CaMKII has been studied. Recent studies done on these autoantibodies reveal some association between neuropsychiatric symptoms and the level of these antibodies [37].

Lumbar puncture must be considered to differentiate PANDAS from autoimmune encephalitis that presents as subacute focal CNS findings, unexplained sudden onset of seizures, and CSF +pleocytosis [38]. Neuroimaging studies such as MRI have shown an increased size of basal ganglia structures caudate, putamen, and globus pallidus because of antibody-mediated inflammation leading to the development of post-streptococcal OCD and tic disorder [21,39-43]. Studies have found that this group of children had an increase in the size of caudate and lentiform nucleus bilaterally on PET scans [39]. Children affected with PANDAS have cardiac involvement presenting with systolic murmurs, which require full cardiovascular assessment, electrocardiogram, and echocardiography [30].

PANDAS is a diagnosis of exclusion and the following panel of tests is proposed for the diagnosis and management of PANDAS (Table 2).

**Table 2:** Investigations for PANDAS PANDAS: pediatric autoimmune neuropsychiatric disorders associated with streptococcal infections; ESR: erythrocyte sedimentation rate; CRP: C-reactive protein; ASO: antistreptolysin O; anti-DNase B: antideoxyribonuclease-B antibody; MRI: magnetic resonance imaging [3,36,44]

	Investigations
Routine laboratory tests	Total blood count
	Liver function test
	Renal function test
	ESR, CRP
	Throat cultures
	ASO titer
	Anti-DNase B
To rule out autoimmune etiology	Antiphospholipid antibodies
	Antinuclear antibody
	Lupus anticoagulant
	Celiac test
To rule out autoimmune encephalitis	MRI
	Lumbar puncture
Diagnostic	Electroencephalogram
	ECG
	Echocardiography
Others	Serum iron and ferritin levels
	Thyroid function test
	Metabolic panel
	Cytokine level

Differentials and association of streptococcal infections with other psychiatric disorders

The current diagnostic criteria for PANDAS, which was proposed based on the study conducted by Swedo et al., include the presence of OCD/tics, prepubertal onset, episodic exacerbation of symptoms, temporal relation to GABHS, and association with neurological abnormalities [43]. The following conditions can be considered in the differentials for PANDAS:

Sydenham’s Chorea

PANDAS was declared to be a distinctive diagnosis when patients presented with neuropsychiatric symptoms that did not meet those attributed to SC. Although the two do share some common features like having an autoimmune basis due to molecular mimicry, they are fairly different from each other [38]. SC presents with symptoms like choreiform (piano-like) movements, hyperactivity, increased severity of tics, decreased muscle tone, and behavioral problems that include obsessions/compulsions/irritability, slurring of speech, decreased oral fluency, and emotional lability, and is a manifestation of rheumatic fever [45]. Its clinical course is ordinarily not as severe as acute cases and may resolve without treatment as well [46].

Psychiatric Disorders

PANDAS is associated with psychiatric comorbidities as seen in the study done by Swedo et al., which reported emotional lability in 66% of children, personality changes in 54%, and nighttime difficulties were noted in more than 50%. Other diagnoses included separation anxiety disorder, major depression, generalized anxiety disorder, a disorder marked by disobedience and defiance to authority figures, specific phobia, dysthymia, enuresis, encopresis, and conduct disorder [38].

Movement/Eating Disorders

Studies over the past few years have also reported movement disorders like dystonia, tremor, opsoclonus-myoclonus, paroxysmal choreoathetosis, catatonia, pervasive developmental disorder, schizophreniform disorder, body dysmorphic disorder, eating disorders, and also conversion disorder symptoms, found to be manifesting shortly after a streptococcal infection [47-53].

Pediatric Acute-Onset Neuropsychiatric Syndrome (PANS)

PANS is defined as a rapid onset of OCD or eating disorder symptoms and accompanying comorbid symptoms including at least two of the following categories: anxiety, emotional lability/depression, irritability/oppositional defiant disorder, attention deficit hyperactivity disorder (ADHD) symptoms presenting as a decline in school performance, sensory/motor abnormalities, and somatic symptoms including urinary frequency or sleep disturbances [35,54]. As noted from several studies, PANDAS is considered a sub-type of PANS since they have several overlapping features except for the temporal relation to streptococcal infection [35,55,56].

Pediatric Infection-Triggered Autoimmune Neuropsychiatric Disorders

Another disorder affecting a cohort of children with OCD who had a rapid onset of psychiatric symptoms with other contributory infectious agents like varicella, and Mycoplasma pneumoniae apart from GABHS has been coined as pediatric infection-triggered autoimmune neuropsychiatric disorders (PITANDS) [57].

Childhood Acute Neuropsychiatric Syndrome

The diagnosis of childhood acute neuropsychiatric syndrome (CANS) was subsequently extended, with the input from a collection of authors, to an acute childhood onset of PANDAS symptoms with no limitations about infections or autoimmunity. Their differentials include infections including bacterial/viral meningitis, encephalitis, and drug-induced ones, e.g., steroids and antihistamines [22].

Other Disorders With Psychiatric Symptoms

The differential diagnoses for PANDAS can also include other disorders that manifest with psychiatric symptoms: Tourette’s syndrome, OCD, ADHD, depression, bipolar mood disorder, autoimmune encephalitis, neuropsychiatric lupus, and CNS vasculitis [58,59].

Autoimmune encephalitis: the diagnostic criteria for autoimmune encephalitis in a study by Graus et al. [60] include the subacute onset of working memory deficits, altered mental status, or psychiatric symptoms, together with at least one of the following: (i) new focal CNS findings, (ii) new-onset seizures without a history of known seizure disorder, (iii) CSF pleocytosis (WBC >5 per mm^3^), and (iv) MRI features suggestive of encephalitis, along with the exclusion of any alternate causes.

Hence, similarities in neuropsychiatric symptoms make it eligible as a differential diagnosis. One of the main methods of evaluation is CSF studies for antibodies via lumbar puncture. The advantages of this diagnostic tool are as follows: firstly, antibodies relevant for clinical diagnosis are more likely to be present only in the CSF than in the serum [38,61]; secondly, the collection of antibodies in the CSF and serum may vary but those in the CSF are most likely to define the clinical picture and in some cases, like for anti-NMDA receptor encephalitis, may better help track clinical progress [62,63]; lastly, CSF studies are less likely to show false-negative or false-positive results as opposed to the same tests done using serum or cell-based assays.

The fact that remains constant across various studies that have been published with regard to PANDAS is that it is a diagnosis of exclusion, because of its striking similarities to various disorders and its lack of affirmative confirmatory tests, and its basis on retrospective data. Hence, differential diagnosis is done with other conditions analogous to it and its diagnosis requires an integrative approach.

Treatment

The current guidelines for treatment according to the PANS/PANDAS Research Consortium (PRC) include three main pillars. They are:

(1) Antimicrobial treatment

(2) Psychotherapeutic treatment

(3) Immunomodulatory treatment

A few general principles regarding the treatment of PANS are listed below:

(A) Establish diagnosis only after a comprehensive evaluation as a “diagnosis of exclusion” [33].

(B) Prioritize and provide psychiatric medications and behavioral interventions for those symptoms that cause the greatest distress [29].

(C) Consider the usage of antibiotics for therapy as well as prophylaxis [64].

(D) Treat symptoms due to autoimmunity or neuroinflammation with anti-inflammatory or immunomodulatory therapies [65].

(E) Monitor patients at regular intervals to evaluate for effectiveness and modify depending on improvement or worsening of the condition.

(F) Treatment can be weaned off or stopped when symptoms subside. But follow-ups are required, because of the relapsing nature of this disease and may warrant reinitiation of treatment.

Antimicrobial Treatment

The regimen includes penicillin, ampicillin (per OS for 10 days), and benzathine penicillin G (intramuscular once - If patient </=27kg/60lb - 600,000 U, if >27 kg/60 lb - 1.2 MU) for acute streptococcal infections. If allergic to penicillin, other groups like cephalexin, cefadroxil, clindamycin, azithromycin, or clarithromycin may be given (per OS for 5-10 days) [64]. A prophylactic course is recommended for all PANS cases, empirically [66]. Various studies support the therapeutic benefit of this treatment [67-69].

Psychotherapeutic Treatment

This is individually tailored to each child’s presenting symptoms. It is usually specific to whatever neuropsychiatric symptoms the child exhibits, such as OCD, ADHD, and depression. The drugs given may include a wide array of antipsychotics that may or may not be combined with behavioral interventions like habit reversal training or cognitive behavioral therapy [29].

Immunomodulatory and Anti-Inflammatory Treatment

Unless there is documented proof of autoimmune etiology or raised inflammatory markers within the nervous system, this particular therapy should not be implemented regardless of the universal indication for PANDAS treatment. The diagnostic skills are therefore reinforced as being a key step for an adequate and effective treatment [33]. The benefit-risk ratio needs to be considerably favorable from a long-term perspective, and immunotherapy has to be discontinued if the individual remains symptomatic with no significant progress. If the clinician encounters no therapeutic response, he/she may have to acknowledge the idea of the symptoms occurring due to permanent changes to the brain rather than acute transient inflammation [65].

## Conclusions

A variant of acute-onset OCD, PANDAS is hypothesized to be caused by an autoimmune reaction to group A streptococcal infection. There is a scarcity of quality research on PANDAS therapies, and published studies have been found to be prone to bias.

While the underlying infections and inflammatory processes in PANS and PANDAS patients are being treated, the psychological and behavioral symptoms must be addressed concurrently to reduce suffering and increase adherence to therapy. During both the acute and chronic stages of the disease, psychological, behavioral, and psychopharmacologic therapies customized to each child's presentation can provide symptom relief and enhance functioning. Further studies are needed to thoroughly evaluate potential diagnostic and therapeutic methods for PANDAS and other types of OCD with an autoimmune origin.
